# Identification of drug targets for Sjögren’s syndrome: multi-omics Mendelian randomization and colocalization analyses

**DOI:** 10.3389/fimmu.2024.1419363

**Published:** 2024-06-12

**Authors:** Yingjie Bai, Jiayi Wang, Xuefeng Feng, Le Xie, Shengao Qin, Guowu Ma, Fan Zhang

**Affiliations:** ^1^ School of Stomatology, Dalian Medical University, Dalian, China; ^2^ Academician Laboratory of Immune and Oral Development & Regeneration, Dalian Medical University, Dalian, China; ^3^ Shanghai Engineering Research Center of Tooth Restoration and Regeneration & Tongji Research Institute of Stomatology & Department of Oral Implantology, Stomatological Hospital and Dental School, Tongji University, Shanghai, China; ^4^ Salivary Gland Disease Center and Beijing Key Laboratory of Tooth Regeneration and Function Reconstruction, Beijing Laboratory of Oral Health and Beijing Stomatological Hospital, Capital Medical University, Beijing, China; ^5^ Beijing Laboratory of Oral Health, Capital Medical University, Beijing, China; ^6^ Department of Stomatology, Stomatological Hospital Affiliated School of Stomatology of Dalian Medical University, Dalian, China; ^7^ Department of Stomatology, Shanghai East Hospital, School of Medicine, Tongji University, Shanghai, China

**Keywords:** Sjögren’s syndrome, Mendelian randomization, drug target, methylation, gene expression, protein, proteomics, genetics

## Abstract

**Background:**

Targeted therapy for Sjögren’s syndrome (SS) has become an important focus for clinicians. Multi-omics-wide Mendelian randomization (MR) analyses have provided new ideas for identifying potential drug targets.

**Methods:**

We conducted summary-data-based Mendelian randomization (SMR) analysis to evaluate therapeutic targets associated with SS by integrating DNA methylation, gene expression and protein quantitative trait loci (mQTL, eQTL, and pQTL, respectively). Genetic associations with SS were derived from the FinnGen study (discovery) and the GWAS catalog (replication). Colocalization analyses were employed to determine whether two potentially relevant phenotypes share the same genetic factors in a given region. Moreover, to delve deeper into potential regulation among DNA methylation, gene expression, and protein abundance, we conducted MR analysis to explore the causal relationship between candidate gene methylation and expression, as well as between gene expression and protein abundance. Drug prediction and molecular docking were further employed to validate the pharmacological activity of the candidate drug targets.

**Results:**

Upon integrating the multi-omics data, we identified three genes associated with SS risk: TNFAIP3, BTN3A1, and PLAU. The methylation of cg22068371 in BTN3A1 was positively associated with protein levels, consistent with the negative effect of cg22068371 methylation on the risk of SS. Additionally, positive correlations were observed between the gene methylation of PLAU (cg04939496) and expression, as well as between expression and protein levels. This consistency elucidates the promotional effects of PLAU on SS risk at the DNA methylation, gene expression, and protein levels. At the protein level, genetically predicted TNFAIP3 (OR 2.47, 95% CI 1.56–3.92) was positively associated with SS risk, while BTN3A1 (OR 2.96E-03, 95% CI 2.63E-04–3.33E-02) was negatively associated with SS risk. Molecular docking showed stable binding for candidate drugs and target proteins.

**Conclusion:**

Our study reveals promising therapeutic targets for the treatment of SS, providing valuable insights into targeted therapy for SS. However, further validation through future experiments is warranted.

## 1 Introduction

Sjögren’s syndrome (SS) is a refractory autoimmune disease pathologically characterized by progressive destruction of exocrine glands, involving several systemic organs such as the oral cavity, eyes, kidneys, liver, lungs, joints, and nerves (1). SS is associated with a significantly higher incidence of non-Hodgkin’s lymphoma compared to other autoimmune disease, making it one of the diseases closely associated with malignancy (2, 3). The efficacy of drugs such as lubricants, glucocorticoids, and immunosuppressants, which are commonly used in the clinical treatment of SS, is not always effective and there is a certain degree of adverse reactions, such as local allergies, gastrointestinal damage, and skin lesions (4). Therefore, exploring drug targets for the treatment of SS is of far-reaching clinical significance and can provide theoretical support for the development of new drugs for the treatment of SS.

Finding drug targets through genetic means can not only greatly improve the efficiency of drug development but also save a lot of human and material resources (5, 6). In addition, proteins, as key regulators of molecular pathways, have widely emerged as a major source of drug targets (7, 8). It has been demonstrated that disease-related protein drug targets supported by genetic associations have a higher likelihood of gaining market approval (5). Therefore, constructing drug targets based on genetic information is a more effective approach to developing drugs.

Mendelian randomization (MR) analyses, which utilize genetic variation as an instrumental variable to enhance inferences about causal relationships between exposures and outcomes, have been widely employed in drug target development and drug repurposing. In contrast to observational studies, MR circumvents the influence of environmental and self-adoption factors because genetic variants are randomly allocated at the time of conception. With advancements in high-throughput genomic and proteomic technologies in plasma and cerebrospinal fluid, MR-based strategies have facilitated the identification of potential therapeutic targets for numerous diseases such as inflammatory bowel disease, multiple sclerosis, and colorectal cancer (9–11). In this study, we systematically identified molecular signatures of genes associated with SS risk by integrating DNA methylation, gene expression, and protein abundance data, providing comprehensive directions for future research and potential therapeutic targets.

## 2 Materials and methods

### 2.1 Data sources for DNA methylation, gene expression and protein quantitative trait loci

**Figure 1:**
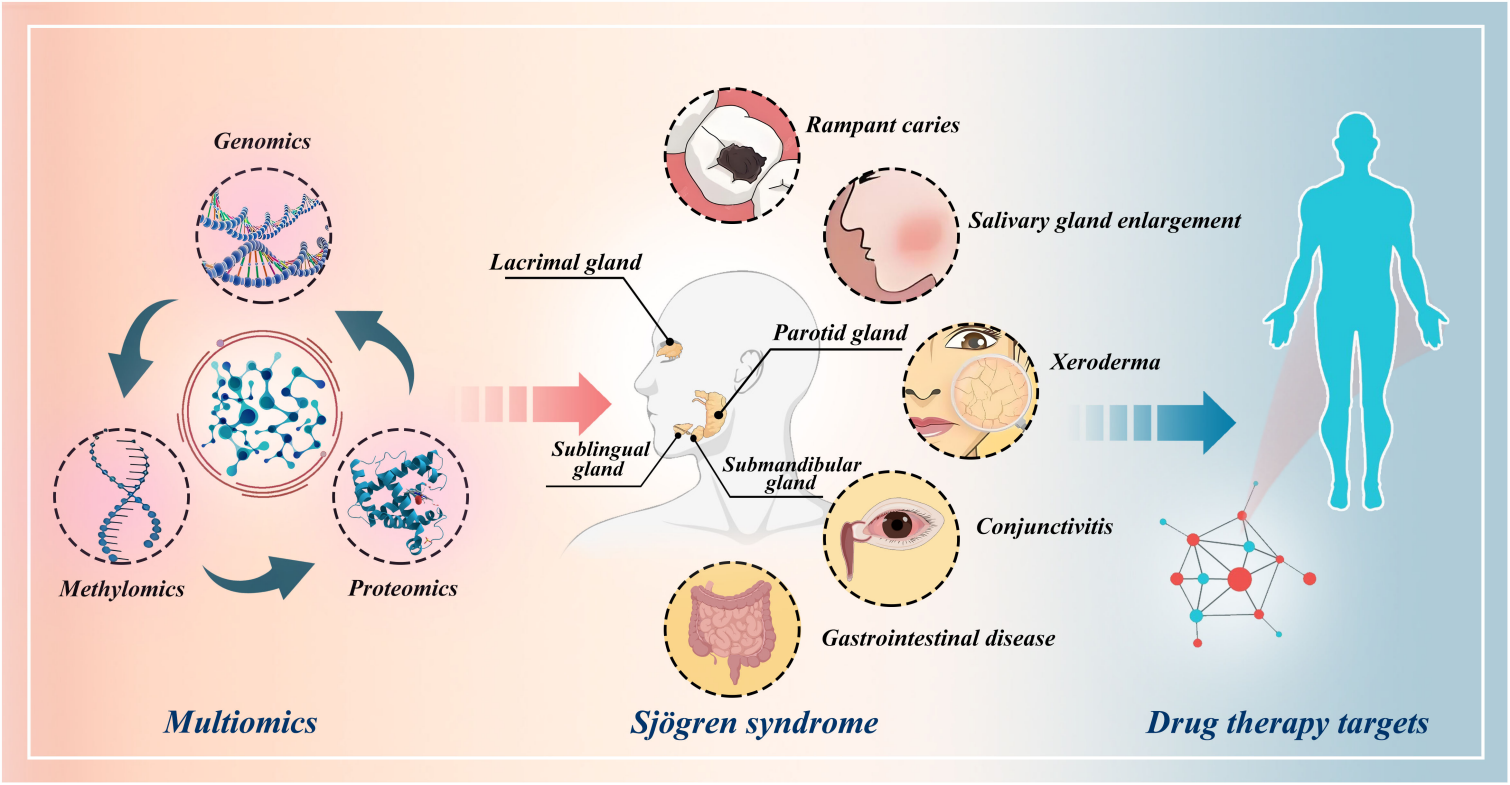
Schematic illustration of the identification of drug targets for Sjögren’s syndrome through multi-omics Mendelian randomization study.

**Figure 2:**
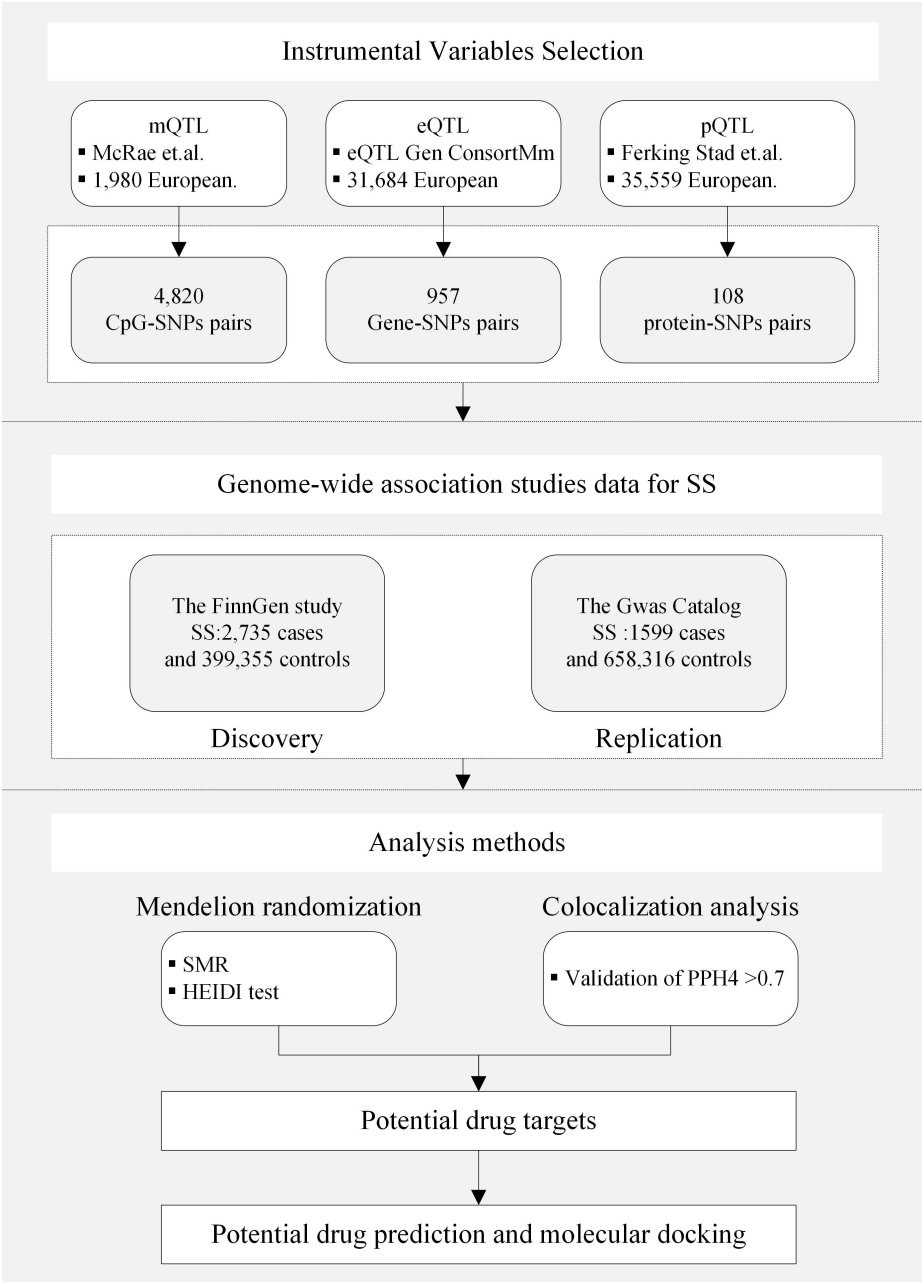
Study design. QTL, quantitative trait loci; SS, Sjögren’s syndrome; SNP, single nucleotide polymorphisms; SMR, summary-based Mendelian randomization; HEIDI, heterogeneity in the dependent instrument; PPH4, posterior probability of H4.

The schematic illustration of the identification of drug targets for SS and the study design is illustrated in **Figures 1** and **2**. Methylated quantitative trait loci (mQTL) data were obtained from SNP-CpG associations in the blood of individuals of European ancestry from 1980 by McRae et al. (12). The blood expression quantitative trait loci (eQTL) dataset was extracted from the eQTLGen consortium (https://eqtlgen.org/), comprising 31,684 individuals, 16,987 genes, and 31,684 cis eQTLs derived from blood samples, primarily from healthy European individuals (13). The protein quantitative trait loci (pQTL) dataset was derived from a large-scale pQTL study of 35,559 Icelanders, with summary statistics extracted for genetic associations at the level of 4907 circulating proteins (14).

### 2.2 SS data sources

Genome-wide association studies (GWAS) data for the SS discovery cohort were obtained from FinnGen Release 10 (https://www.finngen.fi/en). The study was conducted on individuals of European ancestry and comprised a total of 2,735 SS cases and 399,355 control cases. SS patients were identified based on ICD-10 code M35.0, ICD-9 code 7102, or ICD-8 code 73490 (primarily relying on ICD-10 codes). The validation cohort was sourced from the GWAS Catalog GCST90018920 and included 1,599 SS cases and 658,316 control cases (https://www.ebi.ac.uk/gwas/).

### 2.3 Summary-data-based MR analysis

Summary-data-based Mendelian Randomization (SMR) analysis is a statistical method based on the principles of Mendelian randomization that uses genetic variation (single nucleotide polymorphisms, SNP) as an instrumental variable to assess the causal relationship between an exposure and an outcome, and is mainly applied for causal inference between genes and complex diseases or traits, especially when direct randomized controlled trials are not feasible. Compared to MR analysis, SMR analysis relies on pooled results from genome-wide association studies (GWAS) rather than individual-level data, an approach that is more favorable in terms of privacy protection and data sharing. SMR analysis can be combined at the multi-omics level to help researchers explore potential causal relationships between specific drug targets and diseases. In this study, we used SNPs as instrumental variables, mQTL, eQTL, pQTL as exposures, and SS as outcomes. The SMR analysis was conducted using SMR 1.3.1 software (https://yanglab.westlake.edu.cn/software/smr/) (15).

We screened for the top associated cis-QTL by defining a chromosome window centered around the target gene (± 1000 kb) and passing a *P*-value threshold of 5.0 × 10^−8^. The Heterogeneity in Dependent Instrument (HEIDI) test was primarily employed to assess whether a gene SNP-mediated phenotype resulted from a linkage disequilibrium reaction, with the criterion of *P*-HEIDI > 0.01. If the *P*-value of the HEIDI test was less than 0.01, it indicated a heterogeneous association, suggesting possible pleiotropy. A false discovery rate (FDR) of α = 0.05, based on the Benjamini-Hochberg method, was applied for multiple testing. Associations with FDR-corrected *P*-values < 0.05 and P-HEIDI > 0.01 were analyzed for colocalization.

### 2.4 Colocalization analysis

Colocalization analysis can be utilized to genetically co-localize two potentially related phenotypes, determining whether they share common genetic causal variants within a given region. We conducted colocalization analyses to assess whether SS and the identified mQTLs, eQTLs, or pQTLs are influenced by linkage disequilibrium. Five exclusivity hypotheses were examined in the colocalization analyses: 1) No association with any of the traits (H0); 2) Association with trait 1 only (H1); 3) Association with trait 2 only (H2); 4) Causal variants for the two traits are different (H3); 5) Causal variants for the two traits (H4) are the same. For pQTL-GWAS colocalization, eQTL-GWAS, and mQTL-GWAS, the colocalization region windows were set at ±1000 kb, ± 1000 kb, and ±500 kb, respectively. A posterior probability of H4 (PPH4) greater than 0.70 was considered strong evidence for colocalization.

### 2.5 Integrating results at the multi-omics level of evidence

To achieve a comprehensive understanding of the association of gene-related regulation with SS across different levels, we integrated results from three distinct gene regulatory layers. Considering that proteins represent the final expression products of genes and are prime targets for drug therapy, genes associated with SS at the protein level were prioritized as high-quality candidates. Based on this principle, the final candidate genes were categorized into two tiers: 1) Tier 1 genes: These genes were defined as having associations with SS at protein abundance level (FDR-corrected *P*-value < 0.05), PPH4 of colocalization > 0.7, and associations with SS at gene methylation or expression level (original *P*-value < 0.05); 2) Tier 2 genes: These genes were defined as having associations with SS at protein abundance level (FDR-corrected P-value < 0.05), and associations with SS at both gene methylation and expression levels (FDR-corrected *P*-value < 0.05), PPH4 of colocalization > 0.7. Moreover, to delve deeper into potential regulation among methylation, expression, and protein abundance, we conducted MR analysis and colocalization analysis to explore the causal relationship between related DNA methylation and expression, as well as between gene expression and protein abundance.

### 2.6 Candidate drug prediction and molecular docking

Predicting drug candidates through drug targets is a critical step in drug discovery and development. We searched each of the key genes in the DrugBank database to obtain information about the drugs associated with these genes (https://go.drugbank.com/) (16). DrugBank is a comprehensive drug database that contains information about the pharmacological properties, targets, and other information about drugs. DrugBank is often used in conjunction with other databases and tools to explore multi-targeted mechanisms of action of a drug and its potential therapeutic effects.

To further understand the interaction between drug candidates and targets, molecular docking technique was used in this study. The drug structure data and target protein structure data were obtained from the PubChem Compound Database (https://pubchem.ncbi.nlm.nih.gov/), and the Protein Data Bank (http://www.rcsb.org/), respectively (17). We employed semi-flexible docking to form stable complexes. Protein pretreatment (removal of water molecules and excess ligands, addition of hydrogen atoms) was accomplished using PyMOL 2.4. AutoDock Tools 1.5.6 was used to generate PDBQT files for docking simulations. Molecular docking analysis was performed using AutoDock Vina 1.2.2 (http://autodock.scripps.edu/) (18). Binding energies less than -5 kcal/mol were defined to indicate effective ligand-receptor binding, while binding energies less than -7 kcal/mol indicated strong binding activity.

## 3 Results

### 3.1 DNA methylation and SS

A total of 4820 CpG sites were identified as associated with SS risk (*P* < 0.05) (**Supplementary Table S1**). After correction for multiple testing and colocalization analysis, we identified a total of 154 CpG sites associated with SS (*P*_(FDR)_ < 0.05, PPH4 > 0.70) (**Table 1**, **Supplementary Table S1**). For instance, cg18909389 (OR 0.35, 95% CI 0.31–0.41) and cg12257344 (OR 0.33, 95% CI 0.28–0.38), located in CLIC1, as well as cg00355613 (OR 0.27, 95% CI 0.22–0.33), cg15745284 (OR 0.28, 95% CI 0.23–0.34), cg21289669 (OR 0.23, 95% CI 0.18–0.29), and cg07518714 (OR 0.27, 95% CI 0.22–0.34), located in TNXB, were negatively associated with SS risk. Additionally, cg05571472 (OR 6.13, 95% CI 4.33–8.69), located in C6orf48, was positively associated with SS risk. In the validation cohort, many CpG sites such as C6orf25 (cg06132876), PLAU (cg04939496), and TNXB (cg07237769) were replicated (**Supplementary Table S2**).

**Table 1:** Associations of DNA methylation with Sjögren’s syndrome (SS).

Gene	Probe ID	OR (95% CI)	*P* value	PPH4
CLIC1	cg18909389	0.35 (0.31–0.41)	7.75E-46	0.98
TRIM31	cg11100081	0.59 (0.55–0.64)	6.48E-45	<0.01
CLIC1	cg12257344	0.33 (0.28–0.38)	1.67E-44	0.98
TNXB	cg00355613	0.27 (0.22–0.33)	3.72E-36	0.98
HLA-DMB	cg13524037	2.47 (2.14–2.86)	1.69E-34	<0.01
HLA-DPB1	cg14373797	0.8 (0.77–0.83)	2.22E-34	<0.01
C6orf27	cg05239811	0.25 (0.2–0.31)	8.98E-34	0.06
TNXB	cg15745284	0.28 (0.23–0.34)	3.94E-33	0.93
TNXB	cg21289669	0.23 (0.18–0.29)	4.47E-32	0.97
TNXB	cg07518714	0.27 (0.22–0.34)	8.58E-32	0.97
HLA-DPA1	cg05751055	0.51 (0.45–0.57)	1.25E-29	<0.01
TNXB	cg21642103	0.19 (0.14–0.26)	3.37E-28	0.98
TNXB	cg15014577	0.18 (0.14–0.25)	2.29E-27	0.97
COL11A2	cg22122760	0.43 (0.37–0.51)	1.16E-26	<0.01
HLA-DRA	cg08882389	0.18 (0.13–0.25)	1.30E-26	0.12
TNXB	cg11493661	0.17 (0.12–0.24)	1.61E-25	0.98
C6orf48	cg05571472	6.13 (4.33–8.69)	2.08E-24	0.96
CLIC1	cg18402034	0.14 (0.09–0.2)	3.65E-24	0.92
XXbac-BPG308K3.6	cg06608359	0.56 (0.5–0.63)	4.55E-23	1.00
GPSM3	cg21386484	0.31 (0.24–0.39)	8.05E-23	0.78

OR, odds ratio; CI, confidence interval; PPH4, posterior probability of H4.

### 3.2 Gene expression and SS

**Figure 3:**
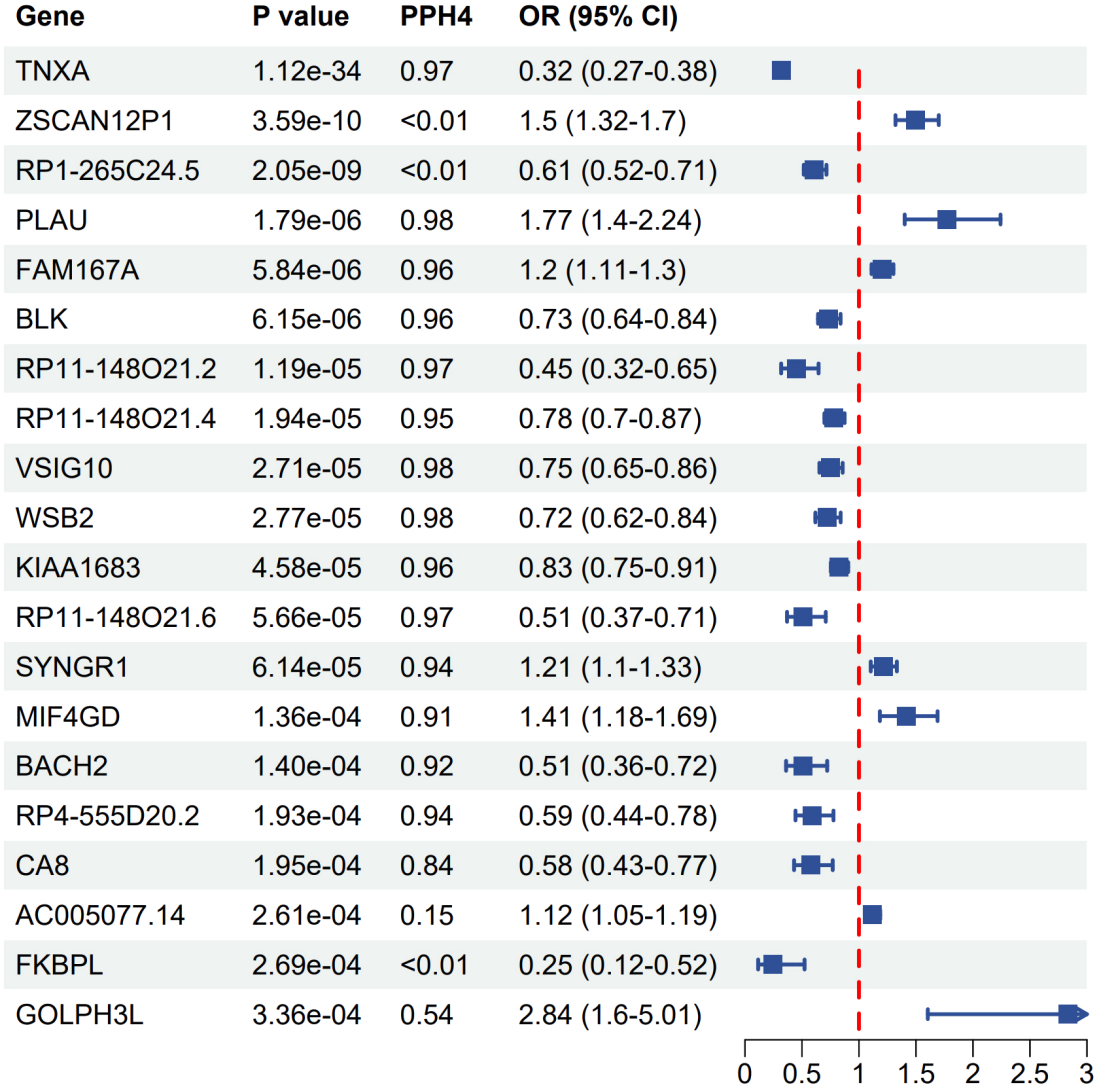
Forest plot of associations between gene expression with SS. OR, odds ratio; CI, confidence interval; PPH4, posterior probability of H4.

A total of 957 genes were identified as associated with SS risk (*P* < 0.05) (**Supplementary Table S3**). After correcting for multiple testing (*P*_(FDR)_ < 0.05) and conducting colocalization analysis (PPH4 > 0.7), genetically predicted CA8 (OR 0.58, 95% CI 0.43–0.77), BACH2 (OR 0.51, 95% CI 0.36–0.72), RP4–555D20.2 (OR 0.59, 95% CI 0.44- 0.78), RP11–148O21.4 (OR 0.78, 95% CI 0.70–0.87), BLK (OR 0.73, 95% CI 0.64–0.84), KIAA1683 (OR 0.83, 95% CI 0.75–0.91), RP11–148O21.2 (OR 0.45, 95% CI 0.32–0.65), TNXA (OR 0.32, 95% CI 0.27–0.38), VSIG10 (OR 0.75, 95% CI 0.65–0.86), and WSB2 (OR 0.72, 95% CI 0.62–0.84) were negatively correlated with SS risk. Conversely, genetically predicted PLAU (OR 1.77, 95% CI 1.40–2.24), FAM167A (OR 1.20, 95% CI 1.11–1.30), MIF4GD (OR 1.41, 95% CI 1.18–1.69), and SYNGR1 (OR 1.21, 95% CI 1.10–1.33) were positively associated with SS risk (**Figure 3**). The associations of FAM167A, BLK, RP11–148O21.2, RP11–148O21.4, RP11–148O21.6, SYNGR1, MIF4GD, and CA8 were replicated in the validation cohort (**Supplementary Table S4**).

### 3.3 Protein and SS

**Figure 4:**
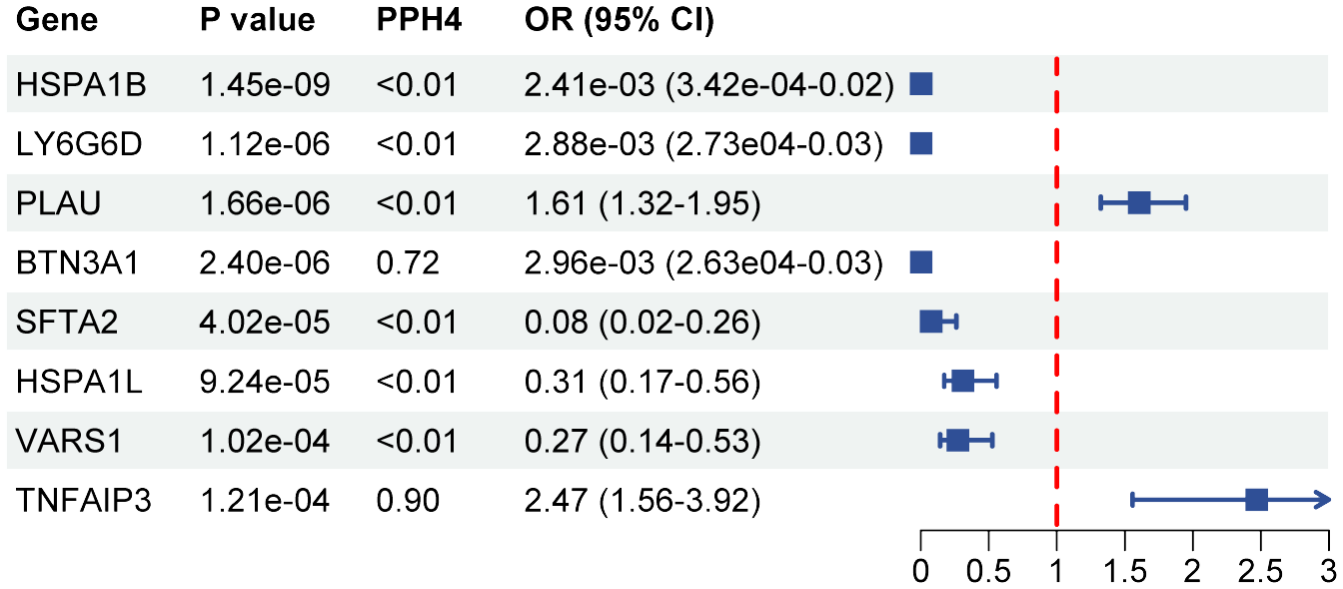
Forest plot of associations between protein with SS. OR, odds ratio; CI, confidence interval; PPH4, posterior probability of H4.

A total of 108 proteins were associated with SS risk at the *P* < 0.05 level (**Supplementary Table S5**). After adjusting for multiple tests, 8 proteins were associated with the risk of Sjögren at the *P*_(FDR)_ < 0.05 level. HSPA1B (OR 2.41E-03, 95% CI 3.42E-04–1.70E-02), LY6G6D (OR 2.88E-03, 95% CI 2.73E-04–3.03E-02), BTN3A1 (OR 2.96E-03, 95% CI 2.63E-04 -3.33E-02), SFTA2 (OR 0.08, 95% CI 0.02–0.26), HSPA1L (OR 0.31, 95% CI 0.17–0.56), and VARS1 (OR 0.27, 95% CI 0.14–0.53) were observed to be negatively correlated with SS risk. Conversely, PLAU (OR 1.61, 95% CI 1.32–1.95) and TNFAIP3 (OR 2.47, 95% CI 1.56–3.92) were positively associated with SS risk (**Figure 4**). The results of the colocalization analysis found high supportive colocalization evidence for BTN3A1 (PPH4 = 0.86) and TNFAIP3 (PPH4 = 0.90). BTN3A1 (OR 0.01, 95% CI 6.31E-04–0.09, P_(FDR)_ = 0.036) was replicated in the validation cohort (**Supplementary Table S6**).

### 3.4 Integrating evidence from multi-omics levels

**Figure 5:**
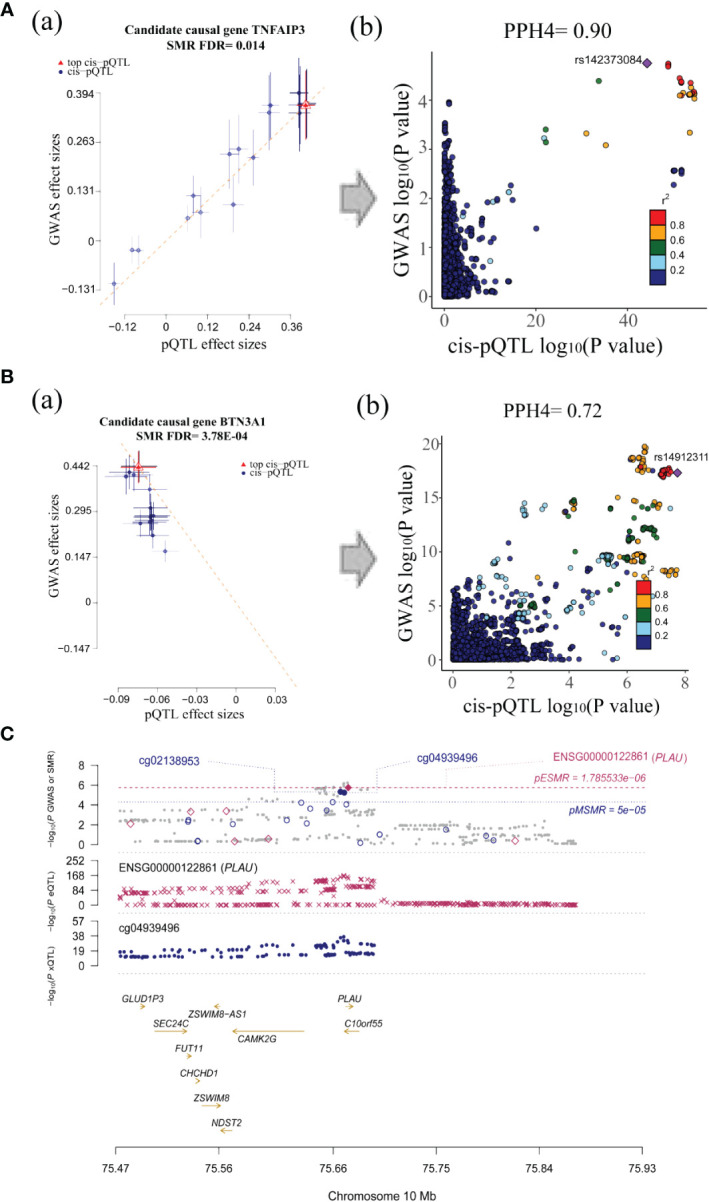
Visualization for associations between candidate causal genes and SS. **(A)** The SMR (a) and colocalization analysis (b) between TNFAIP3 protein and SS GWAS. **(B)** The SMR (a) and colocalization analysis (b) between BTN3A1 protein and SS GWAS (all SMR FDR < 0.05; HEIDI test *P* > 0.01; PPH4 of colocalization > 0.7, the r^2^ value indicates the linkage disequilibrium (LD) between the variants and the top SNPs.). **(C)** Associations between PLAU methylation, expression and SS GWAS.

After integrating evidence at the multi-omics level, we identified 2 tier 1 genes, TNFAIP3 and BTN3A1, and the tier 2 gene PLAU (**Table 2**, **Figure 5**). In the validation cohort, BTN3A1 was replicated at the level of circulating proteins (*P*_(FDR)_ = 0.036) (**Supplementary Table S6**). In exploring the association between gene methylation, expression, and protein abundance, we found that the methylation of cg22068371 in BTN3A1 was positively associated with protein levels, which is consistent with the negative effect of cg22068371 methylation on the risk of SS (**Supplementary Table S7**). Positive correlations were also observed between the gene methylation of PLAU (cg04939496) and gene expression, as well as between gene expression and protein levels, which were corroborated with the positive effect on SS risk. Strong colocalization supportive evidence was observed between the methylation of BTN3A1 (cg22068371) and protein abundance, and between the gene methylation of PLAU (cg04939496) and expression.

**Table 2:** Tier of genetically predicted methylation, expression, and protein of candidate gene with SS.

Gene	Tier	mQTL				eQTL			pQTL		
		Probe	OR (95% CI)	*P* value	*P*_(FDR)_ value	OR (95% CI)	*P* value	*P*_(FDR)_ value	OR (95% CI)	*P* value	*P*_(FDR)_ value
BTN3A1	Tier 1	Cg 22068371	0.47 (0.25–0.88)	0.018	0.570	1.12 (0.96–1.30)	0.163	0.840	2.96E-03 (2.63E04–0.03)	2.40E-06	3.78E-04
TNFAIP3	Tier 1	–				4.35 (1.45–13)	0.009	0.439	2.47 (1.56–3.92)	1.21E-04	0.014
PLAU	Tier 2	Cg 04939496	1.35 (1.18–1.54)	6.73E-06	9.11E-04	1.77 (1.4–2.24)	1.79E-06	4.11E-04	1.61 (1.32–1.95)	1.66E-06	3.19E-04

### 3.5 Molecular docking

**Figure 6:**
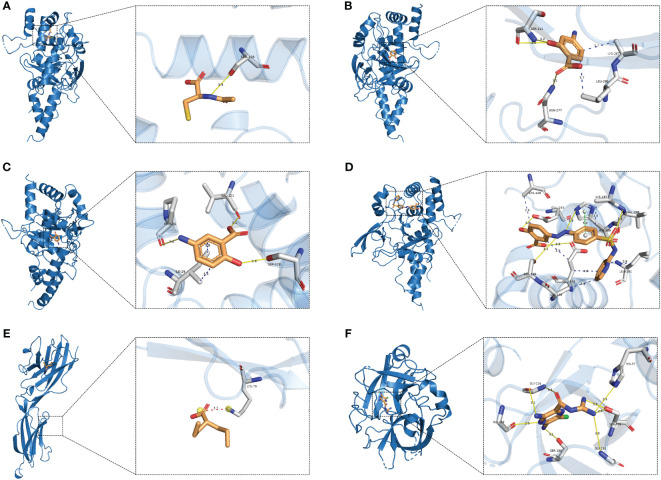
Molecular docking. **(A)** TNFAIP3-Acetylcysteine; **(B)** TNFAIP3-Aminosalicylic; **(C)** TNFAIP3-Mesalamine; **(D)** TNFAIP3-Sulfasalazine; **(E)** BTN3A1-Valproic acid; **(F)** PLAU-Amiloride.

We identified drug candidates related to the target proteins through DrugBank, and the corresponding IDs of drug and protein structure data can be viewed in **Table 3**. The molecular docking of these drugs and proteins encoded by these corresponding target genes was performed using AutoDock Vina 1.2.2. The coordinate of the docking box for protein BTN3A1 was x: y: z= 17.074: -36.189: -7.092. The coordinate of the docking box for protein PLAU was x: y: z= 17.074: -0.176: 18.957. The coordinate of the docking box for protein TNFAIP3 was x: y: z= 20.145: 15.764: 21.938. The drug candidates were attached to their protein targets through hydrogen bonding and strong electrostatic interactions (**Figure 6**). PLAU-Amiloride (-7.4 kcal/mol) and TNFAIP3-Sulfasalazine (-7.3 kcal/mol) had the lowest binding energies and were considered to be the most potential binding mode between ligand and protein.

**Table 3:** Docking results of potential targets with drugs.

Target	PDB ID	Drug	PubChem ID	Binding energy (kcal/mol)
TNFAIP3	2VFJ	Acetylcysteine	12035	-4.2
TNFAIP3	2VFJ	Aminosalicylic acid	4649	-5.0
TNFAIP3	2VFJ	Mesalamine	4075	-5.0
TNFAIP3	2VFJ	Sulfasalazine	5339	-7.3
BTN3A1	4F80	Valproic acid	3121	-1.7
PLAU	1C5W	Amiloride	16231	-7.4

## 4 Discussion

Genes are specific sequences on DNA molecules. They encode proteins or RNAs that regulate gene expression, which can serve as new targets for drug development, i.e., drugs can bind specifically to these molecules, thereby modulating their function or expression. To our knowledge, this study represents the first attempt to utilize MR to identify potential drug targets for SS. We integrated results from multi-omics level evidence, reinforcing the causal relationship between genes and SS risk. Additionally, we combined SMR and colocalization analyses to pinpoint common drivers between potential therapeutic targets and SS risk, while excluding potential confounders. Our study pinpointed TNFAIP3, BTN3A1, and PLAU as potential drug targets for SS. Notably, BTN3A1 was also found to be associated with SS in the validation cohort using a similar analytical approach, underscoring the reliability of the potential drug targets identified in this study.

TNFAIP3 was identified as positively associated with SS risk with high colocalization support. Tumor necrosis factor alpha-induced protein 3 (TNFAIP3) is a crucial nuclear factor κB (NF-κB) regulatory protein that modulates NF-κB expression and apoptosis through multiple pathways (19). Associations between TNFAIP3 and various autoimmune diseases, including SS, rheumatoid arthritis, systemic lupus erythematosus (SLE), and systemic sclerosis, have been documented (16–18). TNFAIP3 has also been identified as one of the susceptibility loci for SS by GWAS (20). Activation of the NF-κB pathway in activated B cells is a key step in the pathogenesis of primary SS (21). The TNFAIP3 gene encodes the A20 protein, essential for the development and functional expression of dendritic cells, B and T cells, and macrophages. The A20 protein serves as a critical negative regulator of NF-κB, and reduced negative regulatory activity of A20 may permit excessive immunoreactivity, leading to increased auto-reactivity (22, 23). Notably, our study found that the top single nucleotide polymorphism (SNP) associated with SS located in TNFAIP3 was rs5029939, which is similar to previous findings that this SNP has been associated with various autoimmune diseases, including SLE, systemic sclerosis, and other autoimmune disorders (24–26). Therefore, we hypothesize that rs5029939 may also be a genetic risk factor for SS susceptibility, although further experimental validation is warranted.

Butyrophilin 3A1 (BTN3A1) is a type I transmembrane protein belonging to the immunoglobulin (Ig) superfamily, with immunomodulatory and antigen-presenting functions. It has been implicated in autoimmune diseases, diabetes mellitus, multiple sclerosis, and cancer (27). Several SNPs, including rs1796520, rs3857550, rs3208733, rs6912853, and rs10456045, of BTN3A1 have been associated with SLE patients (28, 29). Our MR analysis provides evidence that the top SNP rs149123117, located in BTN3A1, is a protective factor against SS, possibly linked to the up-regulation of cg22068371 methylation leading to increased BTN3A1 protein levels.

Plasminogen activator urokinase (PLAU) is a protease involved in fibrinolysis, ECM remodeling, and growth factor activation (30). While most reports on PLAU have been associated with cancers such as breast, colorectal, and esophageal cancers, there is limited evidence of its association with SS. However, in our study, PLAU was found to be associated with an increased risk of SS in terms of gene expression and methylation level. Positive correlations were observed between the gene methylation of PLAU (cg04939496) and expression, as well as between expression and protein levels, supporting the promotional effects of PLAU on SS risk across different regulatory levels.

Our study has some limitations: Firstly, it focused on the relationship between cis-mQTL, -eQTL, -pQTL, and SS, potentially overlooking other regulatory and environmental factors contributing to disease complexity. Although colocalization analysis was used to mitigate bias from linkage disequilibrium, horizontal pleiotropy may still persist. Additionally, the study predominantly involved individuals of European origin, necessitating further research and validation in individuals of other ethnicities for broader applicability. Furthermore, the eQTL dataset derived from blood may not fully capture tissue-specific regulatory mechanisms, warranting further tissue-specific validation. Though molecular docking predicted the interactions of potential drugs and targets, its feasibility may need to be validated by additional *in vitro* and *in vivo* experiments.

## 5 Conclusions

In conclusion, our study identifies TNFAIP3, BTN3A1, and PLAU as potential targets for SS by integrating the potential causal relationship of DNA methylation, gene expression, and protein abundance with SS. These findings provide important insights for targeted therapy of SS, although further experimental validation is required.

## Data availability statement

The original contributions presented in the study are included in the article/**Supplementary Material**. Further inquiries can be directed to the corresponding authors.

## Author contributions

YB: Conceptualization, Data curation, Investigation, Methodology, Software, Writing – original draft. JW: Data curation, Methodology, Software, Writing – original draft. XF: Data curation, Methodology, Software, Writing – original draft. LX: Investigation, Writing – original draft. SQ: Supervision, Writing – review & editing, Conceptualization. GM: Funding acquisition, Supervision, Writing – review & editing. FZ: Funding acquisition, Supervision, Writing – review & editing.
